# BRIDGING ACCESS GAPS: EXAMINING USE OF DIGITAL HEALTH TECHNOLOGIES AMONG SEXUAL AND GENDER MINORITY OLDER ADULTS

**DOI:** 10.1093/geroni/igae098.2195

**Published:** 2024-12-31

**Authors:** Meghan Romanelli, Hyun-Jun Kim, Karen Fredriksen-Goldsen

**Affiliations:** University of Washington, Seattle, Washington, United States; University of Washington, Seattle, Washington, United States; University of Washington, Seattle, Washington, United States

## Abstract

LGBTQ older adults face distinct challenges accessing healthcare. Digital health solutions offer responsive approaches that broaden availability of health information and care, especially for those experiencing isolation, discrimination, and barriers to care. While some LGBTQ older adults prefer digital health technologies, others face difficulties with electronic literacy that prevent use. The current study examined the association of sexual and gender identity and health access factors (barriers to care; health literacy; help-seeking beliefs; healthcare discrimination) with past 12-month use of digital health technologies for communication, insurance matters, and health-information seeking. Data were analyzed from the 2015 healthcare access module of Aging with Pride: National Health, Aging, and Sexuality/Gender Study (NHAS) (N=2,322). Results indicated digital health use varied across LGBTQ older adult subgroups and access factors. Transgender older adults, compared to cisgender, were more likely to use each digital resource to manage their health and healthcare. Participants with experiences of healthcare discrimination more likely communicated with their providers online, while those with lower health literacy turned to the internet to find health information. Older cohorts (65-79; 80+), poverty-impacted, and Black and Hispanic participants were less likely to use digital resources. Digital health technologies may enhance healthcare access for LGBTQ older adults, potentially bridging gaps in service availability and acceptability. This is particularly evident for certain LGBTQ subpopulations such as transgender older adults and those facing greater barriers to care and discrimination. It remains imperative to create digital access and address literacy needs among older cohorts and LGBTQ communities with multiple marginalized identities.

